# Do we all agree what “good health care” looks like? Views from those who are “seldom heard” in health research, policy and service improvement

**DOI:** 10.1111/hex.12528

**Published:** 2017-02-03

**Authors:** Sara Ryan, Jenny Hislop, Sue Ziebland

**Affiliations:** ^1^ Nuffield Department of Primary Care Health Sciences University of Oxford Oxford UK

**Keywords:** focus groups, good health care, health inequalities, qualitative research, seldom heard groups

## Abstract

**Context:**

The aim of this study was to ask whether there are shared ideas about what good health care looks like that apply across different populations and conditions. Do priorities among “seldom heard” groups differ from mainstream views and, if so, how might we understand these differences?

**Design:**

Focus groups were recruited with the help of our study patient representatives. Participants discussed and prioritized a set of eight “core components” of good care. We recorded and transcribed the data for thematic analysis.

**Setting and participants:**

We recruited people who are seldom heard in health and policy research for separate focus group discussions (one each with illegal drug users, Irish Travellers, migrant workers, young men and learning disabled people). We also ran a reference group of educated, older adults and an online group with people with long‐term conditions.

**Results:**

There were few differences in what participants thought was important in health care but considerable differences in their expectations that they might personally receive good care. Differences related to participants' previous experiences. The drug users group reported particularly poor experiences and low expectations of good care.

**Discussion:**

Differences in what is regarded as an entitlement or privilege in health care underline the persistence of structural and relational differences in how services are experienced. While we can be reassured that core aspects of care are similarly prioritized across different patient groups, including those who are seldom heard, a more intractable challenge remains: how to provide equitable health care for marginalized groups in an unequal society.

## 1 INTRODUCTION

Recent attention in the UK has been keenly focused on what goes wrong in health care, and with good reason.^1^, ^2^, ^3^ At the most basic level, health and care services should be safe and delivered by trained staff with the appropriate mix of skills and caseload.^4^ In the last decade, several initiatives have sought shared understanding of what “good care” looks like and how it could be monitored. But we do not know whether these definitions and standards encompass what matters to “seldom heard” groups, that is sections of the population who are typically left out of research.^5^ Are priorities different when care is viewed from less familiar, typically less privileged, perspectives? Given the enduring health inequalities in England (despite a National Health Service free at the point of delivery^6^), a focus on “seldom heard” groups is important. Here, we examine the reach of current ideas about what matters to patients, drawing on focus group discussions with people (illegal drug users, Irish Travellers, migrant workers, young men and learning disabled people) who are seldom heard in health and policy research.

Robert and Cornwell^7^ reviewed the literature on what matters most to patients and examined how this was measured in NHS organizations in England. They concluded there is considerable consensus on the elements that matter most. Relational and functional aspects of care were highlighted: relational aspects include listening to and spending time with patients, using accessible language, being treated as an individual, not being labelled, feeling informed and involved about care and treatment options, having access to knowledgeable professionals and continuity of care. Functional aspects include efficient processes, positive outcomes and information about innovative treatments and technologies. A critical interpretive literature synthesis by Entwistle et al.^8^ mapped how skilled and motivated staff can, through their actions and responsiveness to the individual, enable patients to do what they have reason to value being and doing within and beyond health‐care encounters. The authors produced a comprehensive conceptual map based on qualitative literature on people's positive and negative experiences of care.

The interest in specifying what “good care” looks like in the UK was boosted by the Health and Social Care Act (2012) mandate for the National Institute for Health and Care Excellence (NICE) to develop national Quality Standards for NHS care. These Quality Standards, which have been developed for an array of clinical pathways and care settings, draw on the evidence gathered for clinical guidelines. The standards consist of up to 14 statements for each topic and are intended to define what service providers, commissioners and the public should expect from care. These can then be used to monitor the provider's performance against nationally approved standards. During the development process, NICE invites contributions from stakeholder organizations defined as “national patient, service user and carer groups and voluntary organizations, health‐care professional and academic organizations, and commercial organizations.”^9^

The turn to public involvement is increasingly familiar and justified through accountability and as well potential to improve decision making by involving wider perspectives^10^ (although the evidence for this remains under developed^11^). The approach used by NICE, inviting contribution and observation from registered stakeholder organizations, conforms to expectations about public and patient engagement in policy, but concerns are regularly raised about whether those members of the public who are involved are the “right ones” and whether they are (or should be) representative.^12^, ^13^ In health research, learning disabled people, illegal drug users, homeless or Traveller populations are often left out of mainstream studies; they tend to be included when they are the sole focus of a study, or when the focus of the study is on deficit.^14^ These omissions leave our understanding of patients' experiences incomplete as “seldom heard” groups may have different views about what matters in health care. Indeed, Lemert^15^ argues that those living at the margins of society can, by their very position, view mainstream life with a clearer lens than those at the centre. Enduring health inequalities within the UK, whether yet to be explained by behavioural, material or psychosocial orientations,^16^ underline the importance of understanding the health experiences of seldom heard groups.^17^ Therefore, the aim of this study was to ask whether there are core components of good care that apply across different populations and conditions. Do priorities among “seldom heard” groups differ from mainstream views and, if so, how might we understand these differences?

## 2 METHODS

Between January and April 2013, we ran six face‐to‐face and one online focus group to examine whether how and why ideas about “good care” might differ when discussed with participants from social groups who rarely appear in mainstream research. We drew on published health‐care quality frameworks^18^, ^19^, ^20^ and an earlier qualitative secondary analysis of narrative interviews^21^, ^22^ to identify a set of “core components” of good health care to use in the focus group discussions. We deliberately focused on good rather than basic (such as, my doctor is qualified to treat me) in selecting the components.

### 2.1 Sampling

Participants were recruited through our project Patient and Public Involvement (PPI) contacts, personal relationships in the voluntary sector and social media and held in four different parts of England: Liverpool, West Midlands, Oxford and South London. We wanted four different locations for the groups and these areas were selected pragmatically because they were where the different groups were based. Separate focus group discussions were arranged with migrant workers (n6), Irish Travellers (n8), young men (n10), illegal drug users (n13), people with long‐term conditions (n11) and learning disabled people (n5) as well as a reference group of older people (n9) who meet for educational and cultural activities through the University of the Third Age (U3A). Most of the participants were from pre‐existing groups and were familiar with each other.

### 2.2 Informed consent

We took particular care to adapt our consent procedures for the variety of language, literacy and comprehension skills between groups. Through our local contacts, we circulated information sheets and flyers (approved by CUREC ethics committee, [SSD/CUREC1A/11‐278]) explaining what was involved in the study and inviting people to participate. Several of the Irish Traveller group had poor reading and writing skills and we went prepared to audio record consent, and with several local helpers, who were well known to the participants, available to support literacy throughout the session.

## 3 FOCUS GROUP METHODS AND PROCEDURE

We used focus groups to explore the relevance of the candidate core components with people whose views may be under‐represented in the literature and in the archive we used for secondary analysis. Focus groups are an established method for using with “seldom heard” groups,^23^ allowing researchers to pay attention to those who have “little or no societal voice”.^24^ Being among similar others, in a supportive and reassuring environment, can encourage participants to talk openly. Kitzinger^25^ argues that focus group work “ensures that priority is given to the respondents' hierarchy of importance, their language and concepts, their frameworks for understanding the world.” A skilled group facilitator can shepherd the group through discussions of personal or private issues and possible contradictions between accounts.^26^

Focus groups can also bring difficulties less common in an interview.^27^ The cognitive and emotional demands of reflecting on other people's arguments and engaging opposing views can be particularly challenging for people with learning disabilities.^28^ A group in which some members need frequent explanations or translations to fully participate requires particularly alert and sensitive facilitation. Flexibility in the structure and comportment of groups is particularly important when working with participants who may not usually take part in research.

### 3.1 Focus group structure

Each group was held in a venue convenient to participants, agreed with our local contact. The groups lasted between 90‐120 minutes facilitated by at least two members from the research team and, in two cases, the local PPI member in attendance. Introductions and a warm‐up were followed with discussion about what makes good (and not so good) health care. Participants were invited to consider the eight pre‐prepared candidate components of “good care” and order these and any additional priorities. Finally, the groups discussed why they had ordered the priorities in the way they had.

### 3.2 Online discussion forum

We also held an online discussion forum^29^ with 11 patients with long‐term conditions. The conditions (some comorbid) were as follows: Chiari malformation, postural orthostatic tachycardia, Ehlers‐Danlos syndrome, systemic onset juvenile idiopathic arthritis, cerebral palsy, gender dysphoria, chronic pain, endometriosis. MS, epilepsy, clinical depression, asthma, polycystic ovaries, sickle cell, chronic heart failure, atrial fibrillation, osteoarthritis, cataracts and spina bifida.

A webspace “Goodhealthcare” was set up using Ning, a free online platform for creating social networks. A forum was created for each of the core components. The details of the forum were shared on social media, and eleven people were recruited. We invited participants to read the statements, watch a short illustrative clip from the HERG data archive and then contribute to a discussion about the importance of the statement. Participants were encouraged to return to the webspace to respond to comments left by others. The method allowed us to hear the views of those we could not reach through the focus groups, either because of the severity of a long‐term condition or that of someone they cared for. The facilitator, SR, responded to comments and invited further response which generated richer detail. The site remained open for comments for a six‐week period and then the responses were collated and analysed alongside the other six groups.

All participants were given £30 shop vouchers for their time as well as travel expenses if applicable. Compensation is routinely offered in focus groups.^30^ It helps to increase participation rates and there is some evidence that it does not affect responses.^31^

### 3.3 Analysis

All of the discussions were audiorecorded; the research assistant and facilitators also made notes. The research team met for an analysis workshop in which we listened to the recordings to capture the richness of the interaction between participants. Recordings were also transcribed to enable us to further analyse the content of what was said. An interpretative, thematic analysis was conducted while listening to the recordings and further developed using the transcripts. Our analysis sought to examine and conceptualize the limitations and reach of components of good care and how they may vary between groups. We charted the ordering of the “core components” from the groups which helped to highlight differences and similarities. It also enabled us to explore how participants, referring to their own experiences, regarded the components as good, basic or aspirational.

## 4 FINDINGS

In this section, we compare perceptions and priorities about the constituents of good care between the focus groups. We also consider why priorities and expectations differ, drawing examples from the sorting exercise and transcribed discussions.

### 4.1 Ranking the components of good care

Table 1 shows the rankings of the eight components from six of the focus groups. There are evident similarities, especially in the most highly rated components. Four of the six groups selected “*taking time to answer my questions and explain things well”* as their top priority, while the “drug users'” group chose “*guiding me through difficult conversations”* and the learning disability group chose “*letting me see the same health professional.”* Before further discussion of these data, we should note that some of the participants (particularly in the older adults, Irish Travellers and young men's groups) thought that all of these components could be regarded as essential rather than “good” care. As one of the Irish Travellers put it, when considering priorities

**Table 1:** Focus group exercise, ordering the components (from most to least importance)

Focus group 1 Learning disabled people	Focus group 2 Migrant workers	Focus group 3 Drug users	Focus group 4 Young men	Focus group 5 Irish Travellers	Focus group 6 U3A members
Letting me see the same health professional	Taking time to answer my questions and explain things well	Guiding me through difficult conversations^a^	Taking time to answer my questions and explain things well	Taking time to answer my questions and explain things well	Taking time to answer my questions and explain things well
Involving me in decisions about my care	Guiding me through difficult conversations	Involving me in decisions about my care^a^	Guiding me through difficult conversations	Pointing me towards further support	Pointing me towards further support
Having a friendly and caring attitude	Having a friendly and caring attitude	Letting me see the same health professional	Pointing me towards further support	Having a friendly and caring attitude	Having a friendly and caring attitude
Having some understanding of how my life is affected	Pointing me towards further support	Taking time to answer my questions and explain things well	Having a friendly and caring attitude	Efficient sharing of my health information across services	Efficient sharing of my health information across services
Pointing me towards further support	Efficient sharing of my health information across services	Having some understanding of how my life is affected	Efficient sharing of my health information across services	Guiding me through difficult conversations^a^	Involving me in decisions about my care
Taking time to answer my questions and explain things well	Letting me see the same health professional	Pointing me towards further support	Involving me in decisions about my care	Letting me see the same health professional^a^	Letting me see the same health professional^a^
Efficient sharing of my health information across services	Involving me in decisions about my care^a^	Having a friendly and caring attitude	Having some understanding of how my life is affected	Involving me in decisions about my care	Guiding me through difficult conversations^a^
Guiding me through difficult conversations	Having some understanding of how my life is affected^a^	Efficient sharing of my health information across services	Letting me see the same health professional	Having some understanding of how my life is affected	Having some understanding of how my life is affected

a Shared positions.

But they're **all things that should be there anyway** …. They are all needed, they are all important. [FG5 participant 3]

### 4.2 Differences and similarities in priorities between groups

While most participants agreed that staff “having a friendly and caring attitude” was important, it was apparent from discussions that this included different elements such as displays of empathy (warmth, eye contact, smiling, remembering personal details), behaving with respect, willingness to listen, being careful to check understanding and taking concerns seriously. The migrant workers explained that language difficulties could be overcome if the health professional had a genuine desire to understand. The older adults warned that offence could be caused if staff equated friendliness with overfamiliarity, for example if they used first names without permission.

“Guiding me through difficult conversations” was rated as particularly important by the migrant workers, drug users and the young men, while signposting to further support was rated highly by the older adults and Irish Travellers. Learning disabled people, migrant workers and some of the Irish Travellers said that receiving leaflets or website addresses for additional information would be of little use to them. However, personal advice and information was appreciated. The young men and people with long‐term conditions welcomed additional information from doctors as long as this was not seen as a way to avoid engagement during the consultation. One young man recounted a consultation about a skin problem where the GP hardly spoke and just printed a page from a website for him. People with long‐term conditions said it was important for health professionals to consider broader needs for information and support and wanted details of support and advocacy organizations. The drug users group had different views on this topic depending on whether they were already integrated into support networks, or had not (yet) accessed support from social care or voluntary agencies.

Efficient sharing of information across services was considered important but did not attract any “top 3” ratings. Several participants said one would expect services to be up to date about a patient's health so this was a feature of basic rather than “good” care. However, with the exception of the migrant workers, participants in all groups shared stories about something going wrong because information had not been communicated between services, or between different members of staff in the same service. Young men, older adults and the long‐term conditions groups suggested the best strategy to avoid such failings was to take responsibility themselves for making sure information was communicated where needed. They acknowledged this would not be easy for everyone.

Participants across the groups were clear they wanted to be involved in decisions about their health care, this was in the top 3 priorities for learning disabled people and the drug users groups. Drug users explained that health professionals needed to listen to patients' feedback about the treatment they received and that staff should acknowledge that long‐term drug users often have relevant knowledge about drugs and their effects. Migrant workers said that respect for personal decisions and preferences were particularly important in mental health and women's health care. A slightly discordant note came from the young men who agreed they would lose confidence in their GP's medical abilities if he or she invited them to look at the Internet together during the consultation. They explained that since they would not go to see the GP without “googling” their symptoms first, this was a waste of time for both parties.

There were differences of opinion about whether it was important to “see the same health professional.” Learning disabled people and drug users rated this in their top 3 priorities and said that they valued being able to build a trusted relationship with a doctor. The older adults and Irish Travellers said that seeing the same doctor could be efficient and inspired confidence if it meant that the doctor knew them and their medical history. Some hoped that seeing the same person was less important than having a joined up system

If information is shared and people are talking to me and trusting me then it's not so important to see the same person all the time. [FG5 participant 1]

The young men (who had had few health problems themselves) saw this differently and valued prompt access over personal continuity:

That's not important at all…. If you've got an illness that bad, if you've got an illness and you want to see the same health professional you're not really ill, you just want to have a conversation with somebody. [FG4 participant 7]

There were different views about how important it is for the health professional to understand how the patient's life is affected. Three groups rated this least important and none of the groups rated this in the top 3, although some of the learning disabled people thought that this should be considered basic, rather than good care. One of the reasons for the differences might be the examples that were given, including awareness of people's hobbies and activities. As one of the drug users group put it:

Until you get your main core of problems worked out, you ain't going to be talk about jobs and hobbies. I have to think about whether my tent is going to get flooded and feeding my dog. Nothing like work and hobbies. [FG3 participant 8]

### 4.3 Other examples of good care

At the start of the focus groups, we asked participants to tell us about what they saw as indicative of good care. This was illuminating. The drug users, who were conscious that health professionals sometimes reacted with hostility and suspicion when they saw their history of drug use, stressed the importance of health professionals listening to their views and attending to their feedback about treatment effects. The Irish Travellers said they would like to see colour‐coded medicines for people who do not read, a coordinator or broker to act as a go‐between with services, and access to a familiar doctor for out of hours care. The older people group emphasized prompt referral to specialists, use of consistent evidence‐based approaches to treatment, and recognition that relatives and friends might also need information. Some of the young men who had been in hospitals with friends or family commented on the bright lights, hard reflective surfaces and alarmingly loud noises; they suggested that hospitals would be healthier places if they were quieter and less threatening. Migrant workers said they valued staff who behaved professionally and respected confidentiality—one told a story about a GP's receptionist in her home country who had disclosed a patient's use of antidepressants to a family member. The group of learning disabled people raised a concern that “efficient sharing of information across services” might involve professionals gossiping inappropriately about their health and circumstances. These concerns were not raised by any of the other groups, perhaps because they regard confidentiality as a fundamental of care.

### 4.4 The good, the basic and the unlikely

There was also variation between groups about what *could and should be* expected from health care. An aspect of care that was described as basic or routine in one group might be seen as good, desirable or unattainable in another. To illustrate this point, we consider two examples: “involvement in decisions about care” and professionals “having a friendly and caring attitude.”

#### 4.4.1 Involvement in decisions about care

The older adults group described this as a basic part of health care; their expectations were broadly in line with a shared decision‐making model^32^, ^33^ in which doctors advise on clinical aspects of a treatment, while patients contribute their preferences and priorities to the discussion. People with long‐term conditions saw active involvement in decision making as essential if treatments are to be effective.

If you don't want treatment (or if it's not explained to me) chances are that I am less likely to comply and take the medication/ do the exercise as prescribed. [FG7 participant 8]

Some participants saw this type of involvement as part of good, rather than basic care. For example, the migrant workers said that they liked the fact that in the UK doctors involved them in choices about treatment and that they could trust that the doctor was not benefitting financially if the patient chose a more costly procedure.

The drug users group agreed that involvement was an important aspect and stressed that health professionals also needed to listen to their views:

P3 Involve me in decisions not just for substance misuse but medications in general. They don't listen to whether it's working or not. Not just doctors or nurses but dentists too.

P4 Stigma comes along as soon as you mention drug use. [FG3 participants 3 and 4]

Unlike the other groups, drug users drew on their experiences to show that they did not expect health professionals to listen to them, or involve them in decisions about treatment. They gave several examples in which they felt that health professionals had demonstrated inadequate knowledge about pharmacology. This was contrasted with the comfortable familiarity with drug interactions, contraindications and mechanisms of action displayed by the group participants. However, they knew that as an illegal drug user their requests for pain relief were routinely regarded with suspicion by the professionals because they are treated as if (as one participant put it)

…they've all got ulterior motives, they're all trying to blag this and that and the other, and it's a really easy default argument to fall back on and because [er] some people are professional doctors and some people are service users, are drug addicts, then it's obvious which ones are going to get listened to and which ones who are going to be respected and it's as simple as that really. [FG3 participant 3]

#### 4.4.2 A friendly and caring attitude

Participants gave many examples demonstrating why a friendly attitude, from reception staff as well as clinicians, really matters in health care if the patient is to feel able to discuss sensitive health issues. A friendly caring and warm attitude was commonly presented as an integral component of care, not an optional extra. Young men, older adults and the long‐term conditions groups all raised concerns about the danger of “efficiency” being achieved at the cost of empathy. However, during a discussion about whether staff remembering personal details about the patients was important, a member of the online group said

I can't imagine personal care like this but would love it! The nurses who I've seen in hospital have not been interested in you as a person at all and are so busy that they don't have time to chat with you. It would make you feel so much more cared for and therefore give you strength to manage if you felt that they were interested in you as a person. [FG7 participant 10]

A participant in the drug users group raised an example where the behaviour of health professionals was described as “appalling” after it was revealed that a woman in labour was on a Subutex (methadone) script (see Box 1). Based on their experiences, participants in the drug users group did not expect health professionals to be kind and caring but said they would settle for competence:

I don't expect them to be caring about me, I want them to do their job. I'd rather have someone who is cold and clinical who can do a good job. [FG3 participant 6]

Box 1 1 I remember an incident of being on the maternity suite at the (local hospital) with someone who was giving birth and they, they were on a Subutex script, so they hadn't used illicit drugs in I think maybe ten years and you know I thought we'd better tell the midwife that this person's on a Subutex script. Now they had no idea what Subutex were, they went away and obviously they went away to find out and when they found out their attitude when they came back was appalling. I mean this person hadn't touched drugs in…illicit drugs, in ten years. They put someone at the end of the bed to watch, watch her from then on and it was stupid, it was very stressful, it was upsetting, it was awful. I complained and, and we, we got some kind of I don't really know, an excuse of an apology but it was just the lack of knowledge and ignorance with the, with the midwife, the maternity suite staff. And they, they should be aware of these, these, these sort of things, I thought it was, it was disgusting.

## 5 DISCUSSION

This study presents empirical data about what members of “seldom heard” groups see as components of “good care.” Given enduring health inequalities, we anticipate that these groups may have different priorities and different views of what constitutes “good” care. It is also possible that marginalized people may offer a different, perhaps clearer, view of the mainstream. While the growing consensus on the aspects of care that matter most to patients derives from both research and informed opinion, little attention has been paid to how perceptions of what is (most) important varies. We have shown that there is considerable agreement on the factors that are associated with good care and that many of these are relational aspects of care.

There were some differences between groups in the ordering of priorities which could often be understood through participants' prior health‐care experiences. Strikingly, while there was general agreement about what matters in health care, what was regarded as normal care by one group (e.g. “involvement in decisions” by the older adults) was regarded as evidence of good care by others and regarded as aspirational by another group. In these comparisons, the experiences and expectations of drug users stood in particularly marked contrast to our reference group of older adults. The “eye of the beholder” influences what is taken for granted and what is regarded as exceptional. This is unlikely to be simply a matter of variation in personal preference, psychological profile or style. Experience shapes expectations and experiences are shaped and constrained by social position including sociodemographic variables such as age, gender, education, occupation and where we live. Health and care professionals have different expectations and relationship with middle class older adults, people with long‐term conditions, young men, learning disabled adults, migrant workers, Irish Travellers and illegal drug users. The differences in what is regarded as an entitlement or privilege in health care underline the persistence of structural and relational differences in how services are experienced,^34^ or what Pease^35^ calls, the “normativity of privilege.” We could, on reflection, suggest that the original decision to focus on “good” health care was based on a normative assumption within the research team that there is a shared understanding of what constitutes “basic” health care. Indeed, the examples of “good” care participants provided outside of the eight core components (including prompt referral and receiving appropriate care for the problem) are examples of what we would consider to be “basic” care.

The study has limitations; for example, despite the best efforts and good contacts of our PPI colleagues, we were unable to get past gatekeepers to recruit very frail elderly people living in residential care. Other limitations are common to focus group designs, for example that, even in carefully facilitated groups, some participants may be reluctant to express their views in front of others. The migrant workers group included different levels of competence with English, which was challenging for the translator. All sessions started with an open discussion about good care, during which many of the accounts cited examples of inadequate care. We are aware that these stories may be more “tellable” than descriptions of care that is all right; we are also aware that people who feel powerless may be trying to balance the books when they relate an atrocity story^36^ rather than simply relating what happened.^37^ Indeed, we find it hard to conceive of any simple factual descriptions of events in relation to health care: thus, the strength of analyses such as ours lies in a comparative and interpretive approach.

There are several implications of our findings for researchers and for those designing service improvements, including considerations for sampling and interpretation of patients' reports of care, and the mix of experience sought to contribute to codesign groups. Our findings may help researchers and policymakers who are tasked with collecting, comparing and interpreting patients' perspectives on their care. We have shown that people from a broad range of “seldom heard” groups have similar priorities about what is valued in health care; thus, survey instruments based on current understandings of the core components of good care are likely to cover appropriate domains. Population characteristics, including familiarity with health and care services, which influence experiences may be hard to discern through conventional socio‐demographic data.

Codesign approaches, in which patients and members of the public are invited to help staff identify problems and plan service improvements, have gained popularity.^38^ Our study suggests that such initiatives may sometimes benefit from involving people with relatively little experience of health services, as well as seasoned patients. Members of the public who bring a fresh eye to services can challenge assumptions and bring new insights. The young men group, with relatively little experience of hospital care, raised issues that may be relatively invisible to those who have been working in, or using, a service for many years. For example, noise may be taken for granted in hospitals, but is not inevitable and its impact could be reduced.^39^ The confidentiality of the consultation may be taken for granted by those who have never had reason to expect it would be otherwise. However, there would be losses if only people who can turn a fresh eye to health service problems were involved—it is sometimes only when people have considerable experience of health care that they start to realize how the system works or become aware of variation in skills and service provision.

This work contributes to a field where there has been little evidence about whether (how and why) patients ideas about what constitute good care may differ for those in “seldom heard” groups. Our study was designed to look at the components of “good care” rather than “basic care”—participants also expected their health care to be safe and that health professionals would be well trained and aware of best treatments.

Exploring the relevance of the core components of good care in the focus groups found there were some differences in what is seen as important about health care in these “seldom heard” groups, and certainly no suggestion of a completely different value system, yet experiences and expectations of these prioritized aspects of care were very different underlining the persistence of structural and relational differences in how services are experienced. Thus, while we can be reassured that the reach of existing outcome measures and experience survey instruments may be robust across different groups of patients, including those who are seldom heard, a more intractable challenge remains: how to provide equitable health care in an unequal society.

## CONFLICT OF INTEREST

The authors have no conflict of interests to declare.
